# In reply to Topkan et al.

**DOI:** 10.1093/oncolo/oyag195

**Published:** 2026-05-20

**Authors:** Wing-Lok Chan

**Affiliations:** Department of Clinical Oncology, School of Clinical Medicine, Li Ka Shing Faculty of Medicine, the University of Hong Kong

We thank Topkan and colleagues for their thoughtful and constructive commentary on our study^1^ and for highlighting the important distinctions between sarcopenia, myopenia, and skeletal muscle index (SMI). Their discussion provides valuable conceptual clarity and aligns well with current consensus definitions.

We fully agree that sarcopenia is a multidimensional skeletal muscle disease involving reduced muscle strength, decreased muscle quantity or quality, and, in more advanced stages, impaired physical performance. Contemporary guidelines, including those from the European Working Group on Sarcopenia in Older People (EWGSOP2),^2^ the International Working Group on Sarcopenia,^3^ and the European Society for Clinical Nutrition and Metabolism Special Interest Group,^4^ consistently emphasize low muscle strength as the primary diagnostic criterion, with low muscle mass serving a confirmatory role and poor physical performance defining severe sarcopenia. Accordingly, sarcopenia should not be defined by muscle mass alone.

It is therefore essential to distinguish SMI from sarcopenia. SMI is a quantitative, imaging-based measure of skeletal muscle mass, most commonly derived from computed tomography and normalized for height.^1^ It reflects muscle quantity rather than function or performance and, by itself, does not establish a diagnosis of sarcopenia. SMI should instead be regarded as one structural component within the broader sarcopenia framework.

For clarity, our study (Chan et al.) evaluated SMI as a body composition parameter and did not equate low SMI with sarcopenia.^1^ We demonstrated that SMI was significantly associated with chemotherapy-related toxicities and overall survival in patients with metastatic gastric cancer receiving first-line chemotherapy.

We agree with Topkan et al. that using sarcopenia and SMI interchangeably may lead to conceptual and methodological problems, including overestimation of sarcopenia prevalence and reduced comparability across studies. Moreover, reliance on SMI alone may overlook patients with preserved muscle volume but impaired muscle quality or strength, such as those with myosteatosis.^5^

Nevertheless, we believe that measuring SMI remains clinically important even when direct assessment of muscle strength or physical performance is not available, particularly in oncology.^6^ SMI provides an objective and reproducible assessment of muscle mass that can be readily obtained from routine staging CT scans without additional cost or patient burden. This is particularly relevant in oncology practice, where standardized assessments of muscle strength or physical performance are not routinely incorporated into clinical workflows or systematically recorded.

In addition, SMI reflects a patient’s biological and metabolic reserve, which is critical during systemic anticancer therapy. Low SMI has consistently been associated with increased chemotherapy toxicity, postoperative complications, and reduced survival across multiple malignancies, independent of functional measures.^7^^,^^8^^,^^9^^,^^10^ In this regard, SMI provides prognostic information that complements, rather than replaces, muscle strength assessment.

Third, measurement of SMI enables identification of “possible sarcopenia,” a potentially reversible stage characterized by low muscle mass in the absence of overt functional impairment. Several consensus groups, including the EWGSOP2 and Asian Working Group for Sarcopenia, recognize “possible sarcopenia” as a clinically meaningful condition.^1^^,^^11^ Early identification in patients undergoing cancer treatment may facilitate timely nutritional, exercise, or treatment-modification strategies aimed at preventing progression to overt sarcopenia and related functional decline.
